# New York Letter

**Published:** 1892-05

**Authors:** Wm. L. Russell

**Affiliations:** 151 East 50th St.; New York


### *CORRESPONDENCE.*

---

New York, April 15, 1892.

SYPHILIS.

Several questions relating to the management and history of this mysterious and interesting disease came up for discussion at the last meeting of the County Medical Society. Dr. J. A. Fordyce read a short paper on the factors influencing its severity. He did not think it possible in any given case to predict whether the disease would be mild or severe, but certain conditions undoubtedly exercised some influence. Among these was the size of the initial lesion, which, if unusually large, generally indicated that severe secondary manifestations would follow. Certain constitutional conditions also, such as chronic alcoholism, tuberculosis, malaria, rendered the patient less resistive to the malignant effects of the virus. Extreme youth and old age were periods in which severe attacks were very frequent. If the nervous system were deteriorated through hereditary influences, overwork or worry, lesions of that system were more liable to appear.

The possibility of influencing favorably the course of the disease, by constitutional treatment during the first stage, was the second point discussed. The discussion was opened by Dr. E. B. Bronson. He believed that preliminary treatment directed to the hygiene and habits, and to the emunctories of the patient had a decided influence over the future course of the disease. The care of the mouth and teeth might be useful in preventing ptyalism. The treatment of sebaceous disorders also would mitigate the severity of skin eruptions.

He had adopted a plan of treatment which he thought was instrumental in preventing the spread of the virus to the general system. Syphilis was primarily local in nature, and spread to the body through the medium of the lymphatics. Each infected lymphatic gland thus because a nidus from which the poison continued to radiate. With a view of intercepting its progress, he had adopted the method of mercurial inunctions into the area in which were situated the lymphatics most liable to be first affected. If the chancre were on the penis mercurial soap or ointment was rubbed into the skin of the abdomen below the umbilicus, the thighs and the nates, as it was from these regions that the inguinal glands received their supplies. If the chancre were on the finger, the arm and the side of the chest were the sites selected. The objection that constitutional treatment in the primary stage obscured diagnosis, he thought was an admission that the disease was influenced by it.

Dr. E. Fuller said that an occasional error in the diagnosis of chancre was by no means impossible ; he had seen three cases during the past year in which an apparently hard chancre had appeared very soon after exposure, and no symptoms of syphilis had followed. Patients would seldom believe that they had the disease unless demonstrable symptoms were apparent. He thought it better then to omit constitutional treatment unless secondary phenomena appeared. Dr. W. K. Otis differed from Dr. Fuller on this point, claiming that as soon as the diagnosis of syphilis could be fairly made, the specific treatment should be begun. It was not possible to abort the disease, but such annoying conditions as alopecia could be avoided in some cases by the early use of mercury.

Dr. S. Alexander said in regard to the first point of discussion, that syphilis was influenced favorably or unfavorably by certain conditions attending its development. It was liable to be malignant in childhood. It was mildest in countries in which it had long existed. An unexplainable idiosyncrasy in the patient, such as existed toward fevers, was probably the most influential factor in determining the severity of the attack. It was stated by some that the gouty or rheumatic diathesis predisposed to phagedena and suppurative lesions. This has not been his own experience, however, as it had seemed to him that the disease was milder in such subjects. The environment of the patient exercised an important influence, the worst feature of the disease being seen in hospital patients. Diday had observed forty-eight cases which were allowed to go without treatment, half developed severe symptoms, the other half mild only.

In regard to treatment, he thought the question of the use of tobacco and alcohol of some interest. Tobacco was a local irritant, and in the interest of the hygiene of the mouth might have to be avoided. The moderate use of alcohol need not be abandoned, but hard drinking was injurious, locally and generally. Irregular and unsystematic treatment was to be condemned as positively injurious ; large doses of iodide of potassium were harmful if not needed. The same might be said of the indiscriminate employment of the mixed treatment. The nature of the lesion should be definitely determined, and its proper remedy judiciously selected and systematically applied.

Dr. P. A. Morrow said that mercury was poisonous to some, causing stomatitis, gastro-enteritis, anæmia and nervous depression, even when given in moderate doses. The same might be said of iodide of potassium. The early use of mercury was advisable in most cases however, patients thus treated being more liable to escape severe tertiary lesions.

Dr. C. W. Allen was in favor of excision of the chancre or of circumcision; a source of annoyance and infection being thus removed. He believed in early constitutional treatment. It did the patient no harm, in the event of a mistake in diagnosis being made, and by assuming that syphilis was present, exposure of others might be avoided.

Dr. Paul Gibier, of the Pasteur Institute, believed in the use of tonics in the treatment of syphilis, rather than mercury. Those who worked constantly among the fumes of mercury were not unusually exempt from the disease.

### RELATION OF SYPHILIS TO LONGEVITY.

This interesting question was discussed by Dr. O. H. Rogers, a medical examiner of the Equitable Life Assurance Society. He stated that he had consulted the statistics of the company, and had found that among nearly four hundred thousand insured persons, there were between fourteen and fifteen hundred who had admitted at the time of applying for the insurance that they had had syphilis. All had been free of symptoms and had received no treatment for at least five years. The average age at death of all the insured was forty-eight, while that of the syphilitics was but forty-one. It appeared from his investigations that one-third to one-quarter of the longevity was lost in the case of syphilitics. The proportion of death from general paresis among the syphilitics was a point of interest. Among seventeen thousand deaths from all causes, one to one hundred and seventy died of this disease; among the syphilitics, however, the death rate from this cause was one to eighteen.

THE TREATMENT OF PNEUMONIA.

This was the subject of discussion at the March meeting of the Academy. Dr. Janeway was the first speaker. He said that the possibility of aborting pneumonia was denied by some, and as some cases terminated naturally on the third or fifth day, it might be difficult to prove that the treatment pursued had brought about the result. There was always a possibility, too, that the chill was due to some other cause. Still he had seen cases in which abortion seemed to have been accomplished, when treatment was begun in the chill stage. The method pursued by him had for its object, derivation to the surface. A warm foot bath for twenty or thirty minutes, hot drinks, aconite, liquor ammoniæ acetatis, quinine and opium were the agencies which he employed.

When the disease was developed, the treatment involved attention to a number of points. Fever might demand antipyretic measures, if the rapidity of the pulse and nervous phenomona indicated that it was producing injurious effects. Antipyretic drugs should be used carefully and judiciously, and discontinued if any depressing effects were noticed. Phenacetine was as good as any. The management of the heart required great care. He had found digitalis a trustworthy remedy, if not begun too early, and used carefully, at proper intervals. Small doses were usually sufficient. Our aim should be to give just enough, no more. Some cases had been saved by its use.

Strychnine was useful, for the heart and for the nervous system. It had long been used in the form of nux vomica. Caffeine and camphor were other good heart stimulants, which were especially valuable if the stomach were irritable. A moderate dose should first be given, and increased as necessary.

He regarded oxygen as a very helpful remedy, when cyanosis began to appear. He had seen six cases recover, in which three lobes were involved. In one the respirations were fifty, and the pulse a hundred and fifty. The treatment consisted in the moderate use of alcohol, digitalis and oxygen.

If bronchitis were a prominent feature of the disease, counter-irritation might be employed. Pain and restlessness could be best quieted by opium. Congestion of the kidneys, with the appearance of albumen or blood might be due to alcohol. In one instance in which from a pint to two pints of alcohol were being given in twenty-four hours, the case cleared up when digitalis and caffeine were used instead. There could be no rule, however, about the quantity of alcohol; it was necessary to produce the effect. It should not be begun too early, and it was dangerous to give too much lest the bad effects of the remedy be added to the pneumonia.

Dr. W. H. Stimson did not express faith in any special plan of treatment. He would treat symptoms, beginning with the chill, for which he administered spirits of chloroform. If a pronounced reaction followed, aconite in full doses was required, with cupping for pain and great dyspnœa. A combination of stimulants affected the heart more favorably than any one singly; nux vomica, digitalis and strophanthus acted well, and in some cases digitalis and nitroglycerine. Oxygen was valuable, but he had always been afraid of opium; he would give it only in very small doses combined with camphor and perhaps chloral.

Dr. G. B. Fowler thought that it was quite possible to abort pneumonia. His method was by counter-irritation, such as dry cups and mustard. When the disease was developed, little treatment was necessary or desirable. Alcohol should be used only when essential; the food given should be easy of digestion, but not necessarily fluid. He believed in digitalis as a heart stimulant, but it was not possible to lay down any rule for its employment. In one case which recovered sixty drops of the tincture were given every hour for several doses.

Dr. Katzenbach had found strapping with adhesive plaster the best method for relieving pain. WM. L. RUSSELL.

151 *East* 50*th* *St.*

